# Structural elucidation and molecular docking of ferulic acid from *Parthenium hysterophorus* possessing COX-2 inhibition activity

**DOI:** 10.1007/s13205-014-0253-6

**Published:** 2014-10-07

**Authors:** Naresh Kumar, Vikas Pruthi

**Affiliations:** Department of Biotechnology, Indian Institute of Technology Roorkee, 247667 Roorkee, India

**Keywords:** *Parthenium hysterophorus* L., Ferulic acid, Density functional theory, Thermolysis, Molecular docking, Cyclooxygenase-2

## Abstract

In this study, isolation of ferulic acid from *Parthenium hysterophorus* L. followed by the structural characterization using elemental analysis, FT-IR, NMR, ESI–MS and XRD has been carried out. The molecular geometry, harmonic vibrational frequencies and structural parameters were computed by density functional theory with the 6-311G** basis set. Comparisons between experimental and simulated data of spectroscopic analysis and geometrical parameters was accomplished for their statistical validation and the values of correlation coefficient for ^1^H and ^13^C-NMR chemical shifts, bond lengths and bond angles were found to be 0.934, 0.951, 0.943 and 0.961, respectively. The HOMO and LUMO analyses were used to find out the charge transfer within the ferulic acid. Thermal studies were carried out by thermogravimetry (TG), differential thermogravimetric analysis (DTA), and derivative thermogravimetry (DTG) to confirm the effect of temperature upon the ferulic acid. Furthermore, the binding study of the optimized geometry of ferulic acid has been envisioned with cyclooxygenase-2 for its activity inhibition by molecular docking.

**Electronic supplementary material:**

The online version of this article (doi:10.1007/s13205-014-0253-6) contains supplementary material, which is available to authorized users.

## Introduction

*Parthenium hysterophorus* L. is an annual herbaceous, exotic weed which belongs to the asteraceae family. It grows abundantly in agricultural lands, orchards, forest lands, overgrazed pastures, flood plains, wasteland, around residential colonies, railway tracks and along roadsides (Patel 2011). This noxious weed was introduced in India with the contaminated PL-480 wheat, imported from USA in 1950s. Exposure to *P*. *hysterophorus* L. in human manifests the symptoms of hay fever, eczema, skin inflammation, allergic rhinitis, black spots, burning, asthma, blisters around the eye, diarrhea, breathlessness, severe papular erythematous eruptions and choking Maishi et al. (1998), systemic toxicity in livestock by irreparable damage to liver and kidney, and inhibits the liver dehydrogenases in buffalo and sheep (Gunaseelan 1987; Rajkumar et al. 1988). However, *P*. *hysterophorus* L. possesses useful chlorogenic acids such as caffeic, *p*-coumaric, ferulic, vanillic and *p*-anisic and many more which has been exploited for their biomedical and industrial purposes (Ferguson et al. 2005; Kikugawa et al. 1983; Kumar and Pruthi 2014; Meng et al. 2013). Among them, ferulic acid (FA) is a ubiquitous phenolic cinnamic acid derivative, commonly found in the cell wall of commelinid plants, grasses, grains, beans, seeds of coffee, artichoke, and nuts (Rosazza et al. 1995). FA exhibits wide variety of biomedical activities viz. antioxidant, anti-inflammatory, antimicrobial, antiallergic, hepatoprotective, anticarcinogenic, antithrombotic, increase sperm viability, antiviral, increases binding of IgE to peanut allergens, improve the stability of cytochrome c and inhibit the apoptosis induced by cytochrome c (Chung and Champagne 2011; Mori et al. 1999; Kumar and Pruthi 2014; Middleton et al. 2000; Ou and Kwok 2004; Ou et al. 1999;  Toshihiro et al. 2000; Yang et al. 2007). Recent reports proved that FA also acts as a β-secretase modulator with therapeutic potential against Alzheimer’s disease and also improves the structure and function of heart, blood vessels, liver, and kidneys in hypertensive rats (Alam et al. 2013; Mori et al. 2013). Due to the wide variety of functional aspects of FA, there is an urgent need to study its geometry and other structural properties. Further, to study the role of FA in anti-inflammatory reactions, molecular docking of FA and cyclooxygenase-2 (COX-2) has been carried out. The COX-2 is also known as prostaglandin-endoperoxide synthase (PTGS), an enzyme (EC No.: 1.14.99.1) which is responsible for the formation of key biological mediators such as prostanoids (prostaglandins, prostacyclin and thromboxane). The action of most of the anti-inflammatory drugs is due to their binding ability within the active sites of COX-2, preventing the catalysis of arachidonic acid to prostaglandins (Shorrock and Rees 1988). Pharmacological inhibition of COX-2 can provide relief from the symptoms of inflammation and pain. To the best of our knowledge, this is the first report of its kind of statistical analysis of experimental and simulated data for structural parameters and inhibition of COX-2 by FA.

## Experimental section

### Materials

Plant samples have been collected from the area nearby Indian Institute of Technology Roorkee. The Plant was approved by the Forest Research Institute (FRI), Dehradun (Uttarakhand, India) as *P.* *hysterophorus* L. Plants were thoroughly washed with boiled distilled water and their parts such as stem, leaves and flowers have been separated. The samples were kept to become dry in oven at 35 °C for 24 h. Finally, the dried samples were ground to powder for experimental analysis. All the chemicals and reagents used in this study were purchased from Himedia (Mumbai, India) and of analytical grade.

### Extraction and purification

**Fig. 1:**
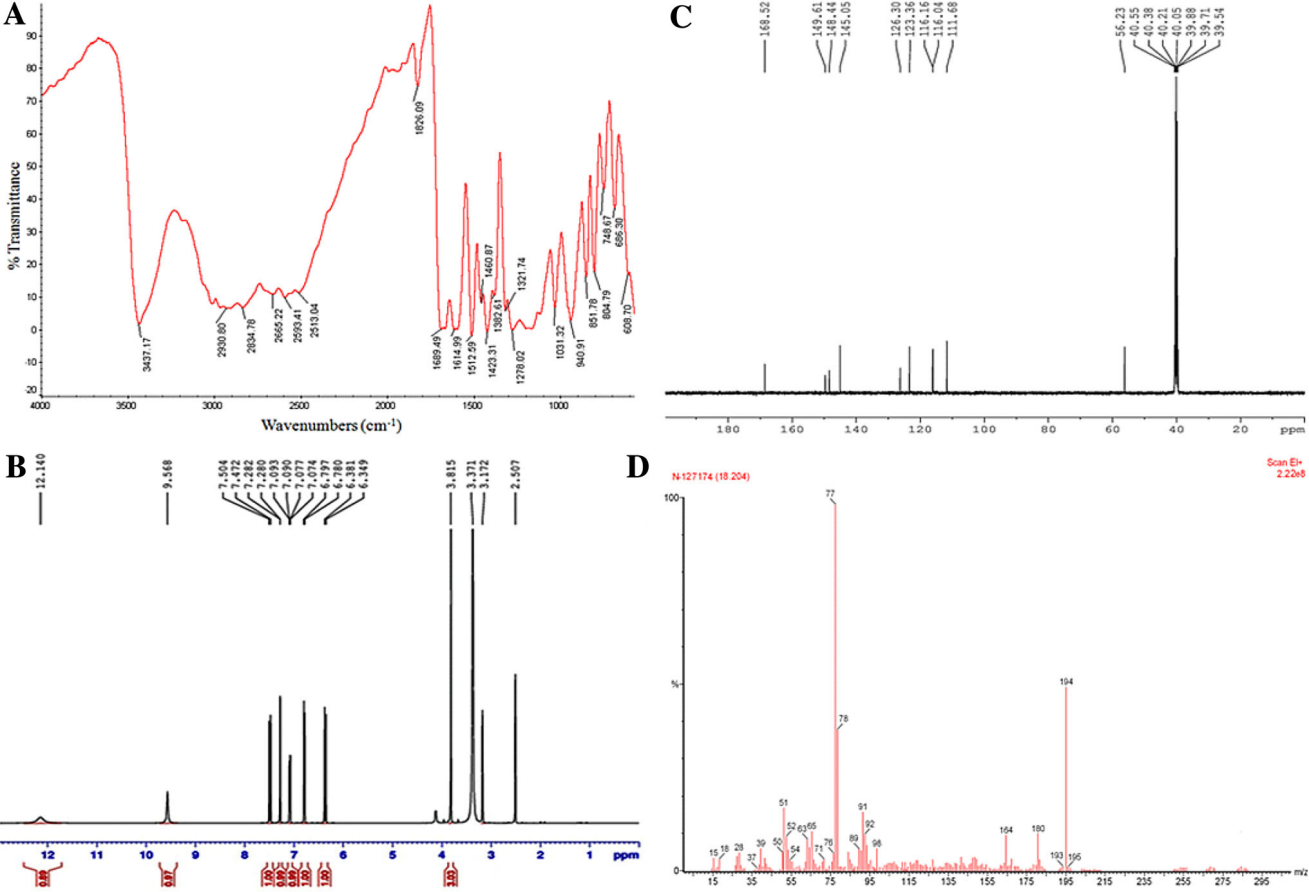
Schematic representation **a** FT-IR spectrum, **b** ^1^H-NMR spectrum, **c** ^13^C-NMR spectrum, and **d** ESI–MS spectrum of FA isolated from *Parthenium hysterophorus* L.

The FA has been extracted by the modifications in the earlier reported method (Tilay et al. 2008). Briefly, 2.0 g of each plant sample (root, stem and leaves) was taken in a 250-ml round bottom flask charged with 60 ml of NaOH (2M). To prevent the oxidation of FA during alkali treatment, 0.001 g NaHSO_3_ was added in each flask and kept within a rotary shaker at 25 °C, 180 rpm for 24 h. Then, all the samples were centrifuged at 12,000 rpm for 10 min and the supernatant so obtained was acidified to a pH of ≤2 by HCl (2 M) solution. The acidified samples were treated three times with 60 ml ethyl acetate and concentrated for the extraction of FA. The concentrated extracts were dissolved in equal volume of acetonitrile/water for further analysis. FA bands from TLC plate were scraped and dissolved in 2.0 ml acetonitrile. The quantitative analysis of all samples was performed on a HPLC column (Merck, Darmstadt, Germany). The isocratic procedure was applied using a mobile phase of acetonitrile/water (80:20) and 0.1 % acetic acid, 20 μl injection volume and flow rate of 1.0 ml/min and analyzed at 320 nm (Zupfer et al. 1998). The melting point of purified FA was found to be 173–175 °C. The FT-IR spectrum of FA (KBr, Fig. 1a) showed the characteristic peaks of carboxylic group (–OH stretching at 3,437 cm^−1^, C=O at 1,689 cm^−1^, C–O at 1,278 cm^−1^), aromatic system (C–H stretching at 3,080–3,030 cm^−1^, combination band at 2,000–1,650 cm^−1^, C=C stretching at 1,600–1,450 cm^−1^, C–H bend at 900–650 cm^−1^), alkene (1,690 cm^−1^) merged with C=O of –COOH group and methoxy group (C–O–C stretching at 1,275–1,200 cm^−1^) also merges with C–O of COOH group. ^1^H-NMR (500 MHz, DMSO-*d*_6,_ ppm) data shown in Fig. 1b depicted the chemical shifts (*δ*) at: 3.815 (*s*, 3H, –OCH_3_), 6.349–6.381 (*d*, 1H, Ar-C8, *J* = 16 Hz), 6.780–6.797 (*d*, 1H, Ar-C5, *J* = 8.5 Hz), 7.074–7.093 (dd, 1H, Ar-C6, *J* = 1.5, *J* = 8 Hz), 7.280–7.282 (*d*, 1H, Ar-C2, *J* = 1.0 Hz), 7.472–7.504 (*d*, 1H, Ar-C7, *J* = 16 Hz), 9.568 (*s*, 1H, –OH exch), 12.140 (*s*, 1H, –COOH exch). The presence of exchangeable protons (phenolic and carboxylic) in FA has been confirmed by adding a few drops of D_2_O in the NMR sample tube. ^13^C-NMR (125 MHz, DMSO-*d*_6,_ ppm) in Fig. 1c indicates the presence of different types of carbon atoms present in FA through their chemical shift (*δ*) at: 56.23 (OCH_3_), 111.68, 116.04, 116.16, 123.36, 126.30, 145.05, 148.44, 149.61 and 168.52 (–COOH). The mass spectrum of isolated compound, shown in Fig. 1d and fragmentation pattern as shown below also confirm the isolated compound as FA.

ESI–MS *m*/*z* 195(M^+^+1, 0.1 %), 194 (M^+^, 53 %), 193 (M^+^−1, 0.1 %), 164 (, 10 %), 94 (, 4 %), 93(, 8 %), 92(, 10 %), 91(, 16 %), 77(, 100 %), 76 (, 6 %), 71(, 4 %), 70 (, 3.8 %), 51(, 18.4 %) and 50 (, 6 %). Elemental analysis calculated for FA (C_10_H_10_O_4_, 194.18) was C: 61.85 %, and H: 5.19, while experimental data revealed C: 61.57 %, and H: 5.09 % for the same.

### Instrumentation

Crystallized acid (FA) was carefully dried under vacuum for several hours prior to elemental analysis on Elementar Vario EL III analyzer. The FT-IR spectrum was recorded on Perkin-Elmer-1600 series FT-IR Spectrometer in KBr pellets. The NMR spectra have been obtained on Avance 500 Bruker Biospin Intl 500 MHz with Fourier transform technique using tetramethylsilane (TMS) as internal standard. Perkin Elmer Clarus 500 gas chromatograph built-in with MS detector was applied for recording the ESI–MS spectrum of FA. Single crystal X-ray diffraction data were collected at 100 K on a Bruker Kappa-CCD diffractometer using graphite monochromated MoKα radiation (*λ* = 0.71070 Å). The empirical absorption corrections were applied in the reduction of data Lorentz and polarization corrections (Sheldrick 1996). The SHELXTL program was used for the structure solution, refinement and data output (Sheldrick 1990, 2000). Non-hydrogen atoms were refined anisotropically, while the hydrogen atoms were placed in geometrically calculated positions using a riding model. The images and hydrogen bonding interactions were created with diamond and mercury (Brandenburg 1999). Refinement parameters and data collection conditions for FA during crystallography experiment are given in the Table 1. Thermogravimetry (TG), differential thermal analysis (DTA) and derivative thermogravimetry (DTG) were carried out using a mass of 0.045 g at 10 °C/min under the nitrogen at 200 ml/min flow rate on a thermogravimetric analyzer (PerkinElmer’s, CA, USA).

**Table 1:** Crystallographic data collection and refinement parameters

Empirical formula	C_10_H_10_O_4_
Color	Yellow
Formula weight (g mol^−1^)	194.18
Crystal system	Monoclinic
Space group	*P 21/n*
*a* (Å)	4.6452 (2)
*b* (Å)	16.8406 (6)
*c* (Å)	12.0347 (4)
*α* (^0^)	90.00
*β* (^0^)	90.139 (2)
*γ* (^0^)	90.00
*V* (Å^3^)	941.45 (6)
Crystal size (mm)	0.31 × 0.27 × 0.23
*Z*	4
*ρ*_calcd_ (g m^−3^)	1.370
*µ*	0.107
*F* (000)	408.0
*θ* Range for data collection	2.08–26.44
Limiting indices	−5 ≤ *h* ≤ 5
	−21 ≤ *k* ≤ 20
	−15 ≤ *l* ≤ 15
No. of measured reflections	1,930
No. of observed reflections	1,581
Data/restraints/parameters	1,930/0/130
*R1^b^ (*I* > 2*σ*(*I*))	0.0413
R1 (all data)	0.0511
*wR2^c^(*I* > 2*σ*(*I*))	0.1469
*wR*2(all data)	0.1575
$${\text{R1 = }}\Sigma ||F_{o} | - |F_{c} ||/\Sigma |F_{o} |,wR2 = \{ \Sigma [w(F_{o}^{2} - F_{c}^{2} )^{2} ]/\Sigma w(F_{o}^{2} )^{2} \} ^{{{1 \mathord{\left/ {\vphantom {1 2}} \right. \kern-\nulldelimiterspace} 2}}}$$	

### Quantum chemical calculations and statistical analysis

The quantum chemical calculations for the geometry optimization of FA were performed by Gaussian 09 with a hybrid function B3LYP at DFT/6-311G** basis set (Becke 1993; Frisch et al. 2009; Peng et al. 1996; Stephens et al. 1994; Goel and Singh 2013). The structural parameters for optimized structure of the FA viz. dipole moment, total energy, bond lengths, bond angles, and HOMO–LUMO energy were computed. The ^1^H and ^13^C-NMR chemical shifts were also calculated by the Gauge-Independent Atomic Orbital method at corresponding basis set. Theoretically computed chemical shifts were converted to TMS scale prior their comparison with the experimental data. The electronic absorption spectra for FA with time-dependent DFT (TD-DFT) in solution (ethanol and DMSO) and gas phase were also computed. Different physico-chemical properties viz. electronegativity (*χ*), chemical hardness (*η*), chemical softness (*S*), chemical potential and electrophilic index (*ω*) for FA were calculated using Koopmans theorem for a closed shell molecule (Koopmans 1934). The experimental and simulated values for chemical shifts (^1^H and ^13^C-NMR), bond lengths and bond angles were statistically tested for their mathematical significance in the MATLAB R2010a toolbox by curve fitting analysis (Goel and Kumar 2014; Kumar and Bhalla 2011). The correlation coefficient (*R*) value which measures the potency and direction of a linear association between two variables was also calculated between simulated and experimental data.

### Molecular docking

In the present work, molecular docking of FA and COX-2 enzyme has been carried out with the help of AutoDock 4.2.3 suite, which uses the genetic algorithm (GA) for its internal conformation search and produces an assembly of conformations by applying Lamarckian genetic algorithm to study the interactions between the ligand and receptor (Morris et al. 2009; Garg et al. 2013). The structure of COX-2 (PDB ID: 6COX) used in this study is available at protein data bank (http://www.rcsb.org). During the docking experiment, water molecules and heteroatoms have been removed, while the hydrogen atoms were added at appropriate geometry groups. The COX-2 was ionized as required at the physiological pH and its protonated form was used for the final docking experiment. The docking parameters used in the present research work are: number of GA runs: 30, population size: 150, maximum number of energy evaluations: 2,500,000, maximum number of generations: 27,000, maximum number of top individuals that automatically survive: 1, rate of genetic mutation: 0.02, rate of crossover: 0.8, GA crossover mode: two points, mean of cauchy distribution for gene mutation: 0.0, variance of cauchy distribution for gene mutation: 1.0, and number of generations for picking worst individual: 10. The simulations were run with a predefined number of generation cycles composed of mapping and fitness evaluation, selection, crossover, mutation, elitist selection steps, and continued with a local search, followed by the scoring of the produced conformers. The energy-based AutoDock scoring function includes terms accounting for short range Van der Waals forces, electrostatic interactions, loss of entropy upon ligand binding, hydrogen bonding, and solvation. The input files (.pdb files) of COX-2 and FA were converted into the subsequent.pdbqt format output files, which contain the necessary parameters for docking such as atom coordinates, partial charges, and salvation. To know the binding sites in COX-2, blind docking was done with the grid size of 126, 126, and 126 along with the *X*, *Y*, and *Z* axis and 0.697 Å of the grid spacing. The center of the grid was set to 29.5, 31.8, and 23.5 Å.

## Results and discussion

### Isolation and purification

Isolation of FA from *P. hysterophorus* L. samples (viz. root, stem, leaves and whole plant) has been carried out by alkali treatment method. The qualitative and quantitative analysis was carried out by chromatographic techniques. The TLC chromatograms of all the samples with commercially purchased ferulic acid (Sigma–Aldrich, India) indicate the presence of FA in each sample when sprayed with 10 % ferric chloride solution (Fig. S1). The HPLC analysis of spots scraped from TLC plates confirmed the purification of FA with retention time of 29.07 min (Fig. S2). The content of FA in whole plant was found to be 123–145 mg/100 g (38–45, 53–60 and 32–40 mg in root, stem and leaves, respectively).

### FT-IR, NMR and mass spectroscopy

As compared to the free carboxylic acid (C=O bond stretching at 1,760–1,690 cm^−1^, O–H bond stretching at 3,000–2,500 cm^−1^, C–O bond stretching from 1,320 to 1,210 cm^−1^, O–H bend from 1,440–1,395 and 950–910 cm^−1^) the C=O stretching has been occurred at 1,689.49, O–H stretching at 2,930.80 cm^−1^, C–O stretching at 1,321.74 cm^−1^, while O–H bending was observed at 1,460.87 cm^−1^ in isolated FA and the shifting of stretching in phenolic O–H (3,550–3,200 cm^−1^) recorded at 3,437.17 cm^−1^. The observed shifting (decrease in wavenumber) in the peaks of functional groups occurred due to the intermolecular H-bonding between FA molecules. The data showed that the chemical shift values of the proton present in carboxylic acid during ^1^H-NMR were found at 12.140, while aromatic protons have been coming into the region of 6.349–7.504 ppm. The fragmentation pattern of the isolated FA during mass spectroscopy also confirmed the structure as 4-hydroxy-3-methoxycinnamic acid (Ferulic acid).

### Crystal structure description

**Fig. 2:**
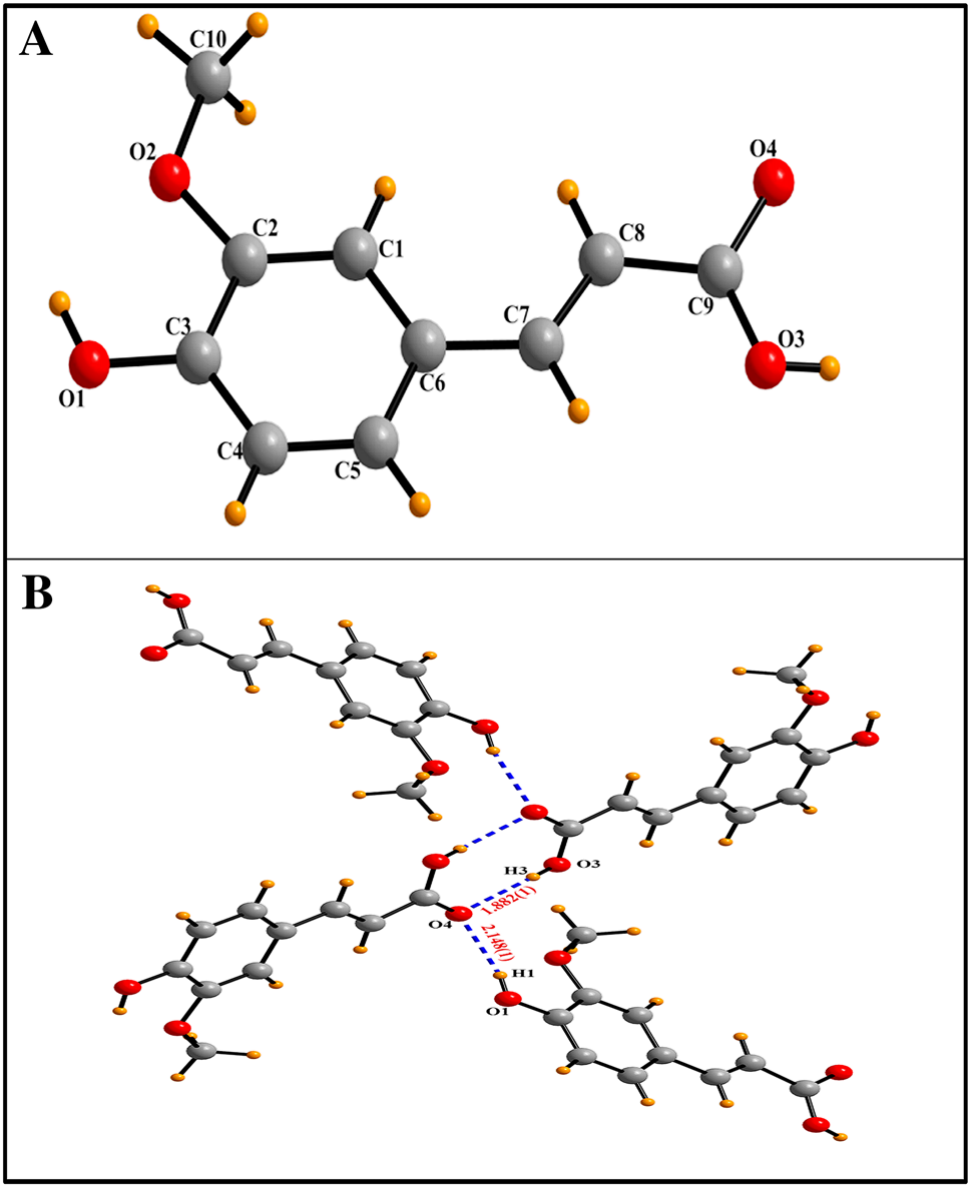
**a** Ball and stick models of the solid-state single crystal structure **b** various O–H···O non-covalent interactions in FA

After spectroscopic characterization of the isolated compound as FA (4-hydroxy-3-methoxycinnamic acid), we crystallize the FA to study its structural properties such as bond lengths, bond angles, inter(intra)-molecular H-bonding and many more. FA was crystallized in the monoclinic space group *P 21/n*. The crystal packing of FA (Fig. S3) showed that the dominant O–H···O hydrogen bond was formed via the interaction of OH of –COOH group of one FA molecule with the oxygen atom of the C=O group of adjacent molecule and vice versa forming a dimer which is responsible for the formation of *R*_2_^2^(8) motif via strong [O_3_–H_3_···O_4_, 1.882(1) Å] non-covalent intramolecular interactions (Table 2). On the other side, the phenolic group (–OH) of FA also exhibited a strong O–H···O [O_1_–H_1_···O4, 2.148 (1) Å] non-covalent interaction with the C=O group of adjacent acid molecule (Fig. 2a, b).

**Table 2:** Non-covalent interactions for ferulic acid [distances (Å) angles (^°^)]

D–H···A	*d* (D–H)	*d* (H–A)	*d* (D–A)	DHA
O1–H1···O4	0.821	2.148 (1)	2.898	152.0
O3–H3···O4	0.820	1.882 (1)	2.637	152.6

### Thermal analysis

**Fig. 3:**
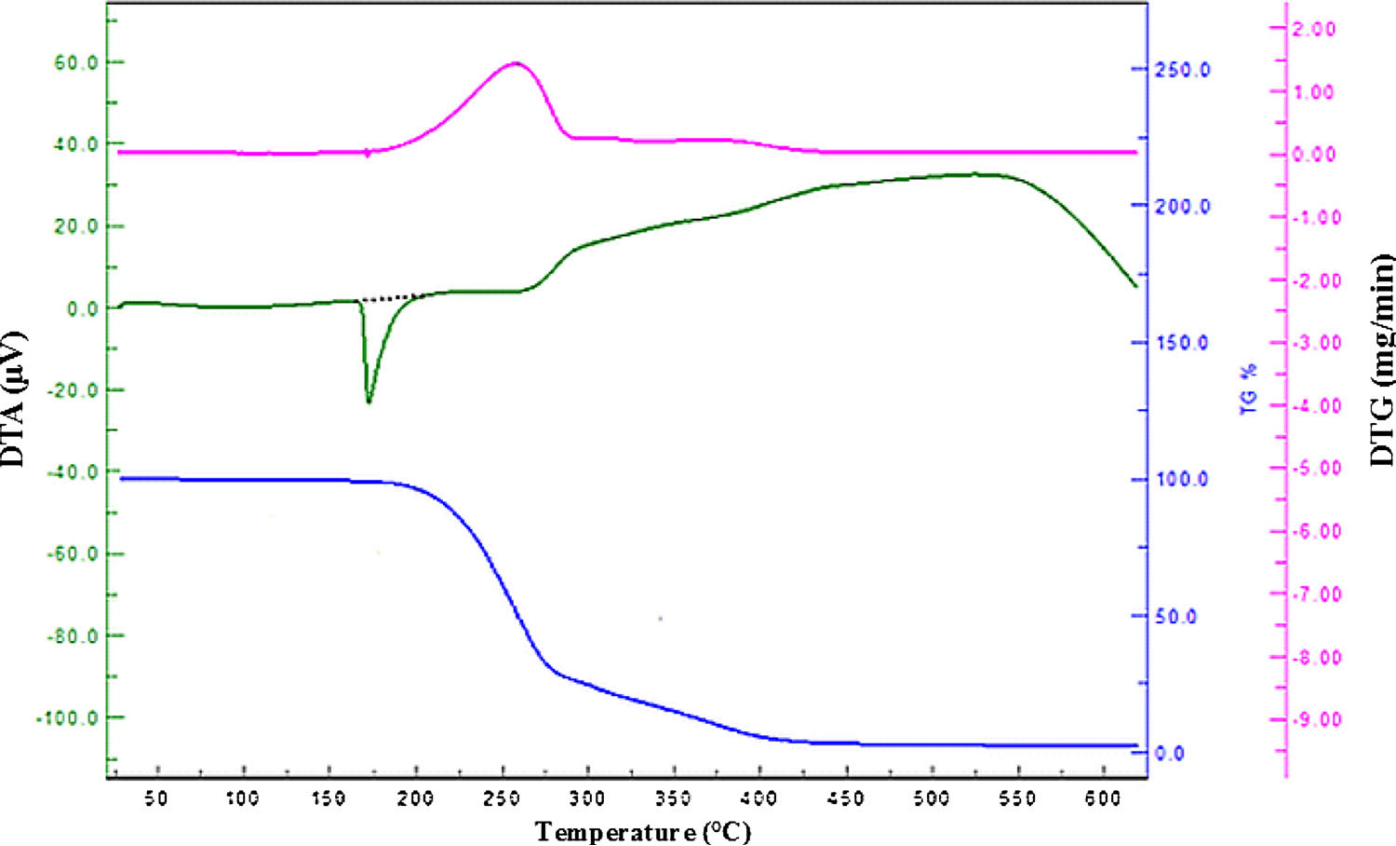
Simultaneous representation of TG-DTA-DTG thermal graphs of FA under nitrogen environment

The thermal stability of FA has been demonstrated by TG-DTA-DTG study. The TGA-DTG curves for FA as shown in Fig. 3 clearly indicated that it was stable up to 100 °C but at higher temperatures curves for thermal study showed irregular pattern. The decomposition of FA occurs in one step in which it leaves out with 99.1 % weight loss (DTG peak at 260 °C) in between 200 and 432 °C, corresponding to endotherm at 173 °C in DTA thermogram.

### Geometry optimization

**Fig. 4:**
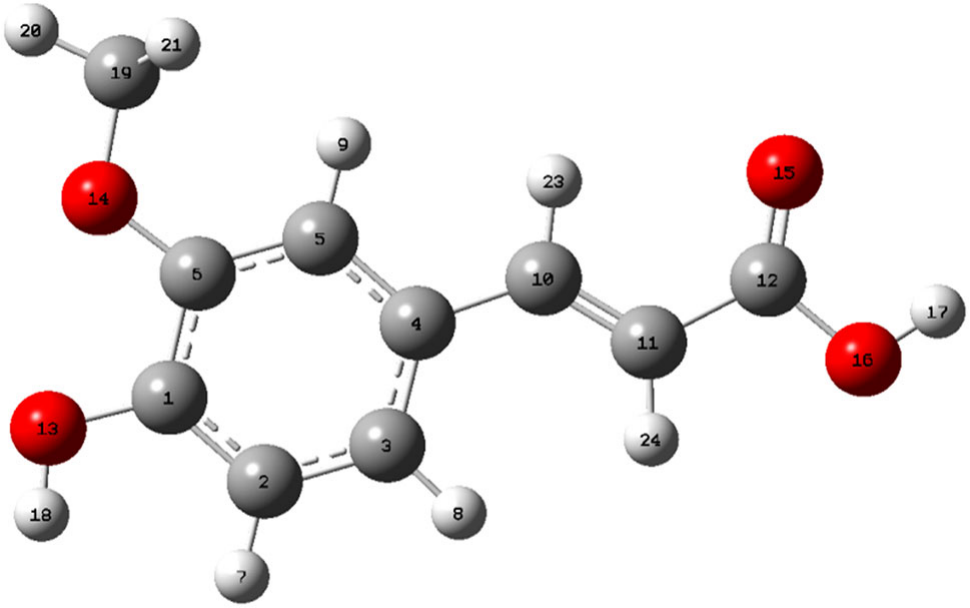
Optimized geometry of FA computed by Gaussian 09 at DFT/6-311G** basis set

**Fig. 5:**
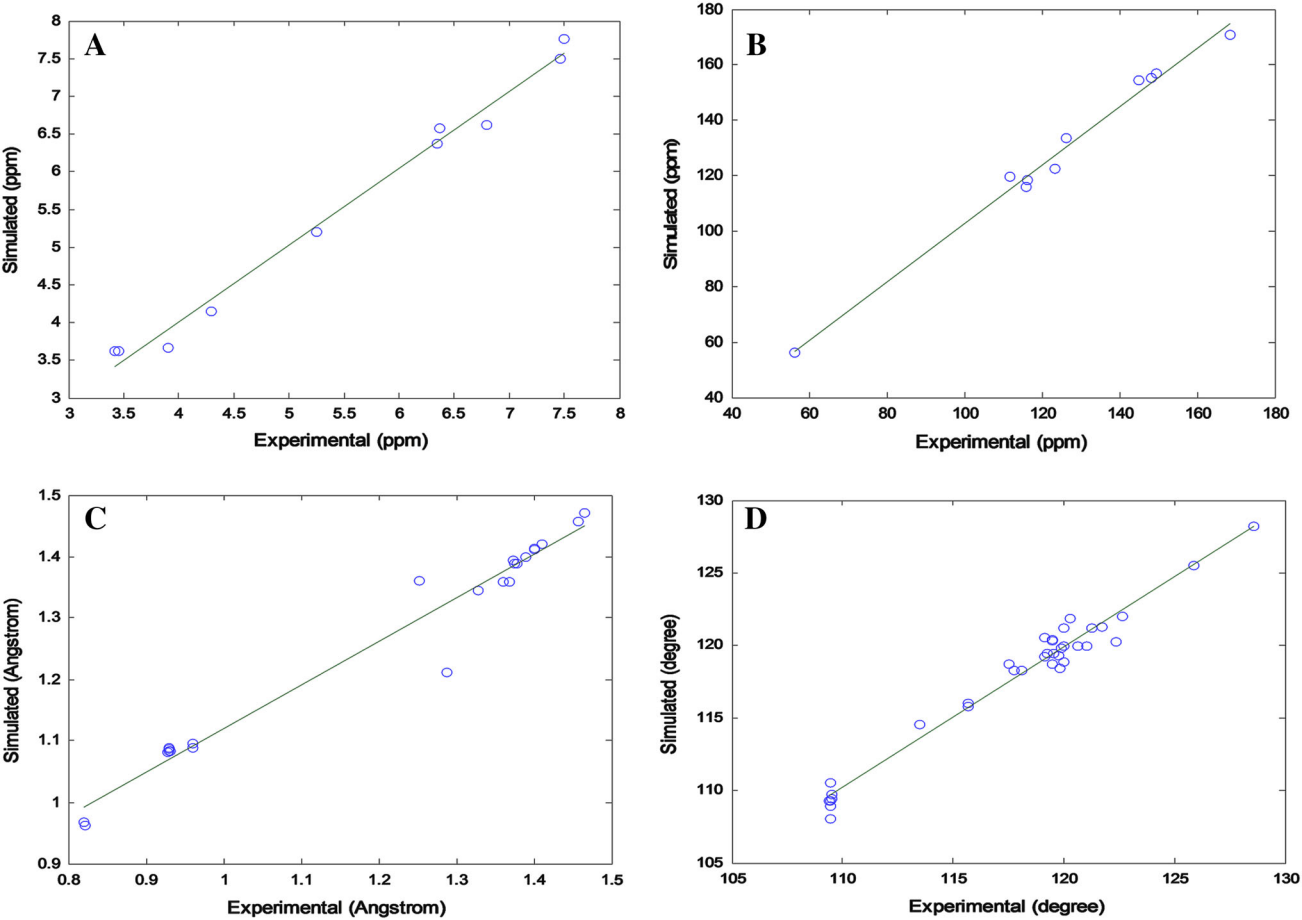
Plots of linear correlation between experimental and simulated values of chemical shifts of **a** ^1^H-NMR, **b** ^13^C-NMR, **c** bond length and **d** bond angle, respectively, for FA

The optimized geometry of FA showed the positive harmonic vibrational frequencies, which indicate that it attained the global minimum on the potential energy surface. Single-point energy calculations and zero-point corrected total energies were also computed. The optimized structure of FA at DFT/6-311G** basis set as shown in Fig. 4 matches with the structure obtained by single crystal diffraction. The comparison between experimental and simulated chemical shifts for proton and carbon is given in Table 3, while the structural parameters such as bond lengths and bond angles are provided in Table 4. From the statistical analysis of data, it was found that the value of correlation coefficient (*R*) for four parameters, ^1^H and ^13^C-NMR chemical shifts, bond lengths and bond angles is 0.934, 0.951, 0.943 and 0.961, respectively. The statistical analysis was performed to validate the theoretical and experimental data. The plots for curve fitting analysis are shown in Fig. 5, which clearly indicate the significant resemblance between theoretical and experimental data obtained from NMR and X-ray crystal structure. Commenting on the statistical results, we inferred that simulated values lie statistically closed to the values obtained from experimental results and outcome of all the four cases to be worthy with respect to their statistical significance for correlation.

**Table 3:** Comparison of ^1^H and ^13^C-experimental chemical shifts with DFT/6-311G** wave function by GIAO method

Proton (^1^H)	DFT/6-311G**	Experimental chemical shifts (ppm)	Carbon (^13^C)	DFT/6-311G**	Experimental chemical shifts (ppm)
7	6.578	6.374	1	156.9466	149.61
8	7.499	7.469	2	118.4485	116.16
9	6.623	6.794	3	122.6049	123.36
17	5.194	5.249	4	133.4492	126.30
18	3.663	3.902	5	119.6773	111.68
20	4.146	4.301	6	155.0316	148.14
21	3.627	3.459	10	154.4157	145.05
22	3.627	3.420	11	115.9099	116.04
23	7.761	7.501	12	170.6272	168.52
24	6.374	6.343	19	56.15	56.23

**Table 4:** Comparison of X-ray structure and calculated optimized structure with DFT/6-311G** for ferulic acid purified from *P*. *hysterophorus* L

Bond length (Å)	X-ray	DFT/6-311G**	Bond length (Å)	X-ray	DFT/6-311G**
O1–C3	1.360	1.358	C4–C5	1.378	1.389
O1–H1	0.821	0.963	C4–H4	0.930	1.086
O2–C2	1.368	1.358	C5–C6	1.389	1.399
O2–C10	1.411	1.421	C5–H5	0.930	1.083
O3–C9	1.252	1.361	C6–C7	1.458	1.457
O3–H3	0.820	0.968	C7–C8	1.328	1.344
O4–C9	1.287	1.229	C7–H7	0.930	1.088
C1–C2	1.375	1.389	C8–C9	1.465	1.471
C1–C6	1.401	1.411	C8–H8	0.931	1.083
C1–H1A	0.929	1.082	C10–H10A	0.960	1.058
C2–C3	1.401	1.413	C10–H10B	0.960	1.046
C3–C4	1.374	1.393	C10–H10C	0.960	1.056

Bond angle (°)	X-ray	DFT/6-311G**	Bond angle (°)	X-ray	DFT/6-311G**
C3–O1–H1	109.47	107.81	C5–C6–C1	118.08	118.29
C2–O2–C10	117.75	118.24	C5–C6–C7	119.55	123.45
C9–O3–H3	109.44	106.06	C1–C6–C7	122.36	118.26
C2–C1–C6	120.30	121.84	C8–C7–C6	128.59	128.19
C2–C1–H1A	119.89	119.77	C8–C7–H7	115.70	115.81
C6–C1–H1A	119.81	118.39	C6–C7–H7	115.71	116.00
O2–C2–C1	125.89	125.51	C7–C8–C9	121.05	119.96
O2–C2–C3	113.48	115.56	C7–C8–H8	119.48	123.34
C1–C2–C3	120.62	118.93	C9–C8–H8	119.46	116.70
O1–C3–C4	119.46	123.36	O3–C9–O4	122.66	122.00
O1–C3–C2	121.28	117.18	O3–C9–C8	119.80	111.28
C4–C3–C2	119.25	119.46	O4–C9–C8	117.54	126.72
C3–C4–C5	120.03	121.18	O2–C10–H10A	109.50	105.69
C3–C4–H4	119.98	118.87	O2–C10–H10B	109.46	111.51
C5–C4–H4	119.99	119.95	O2–C10–H10C	109.49	111.51
C4–C5–C6	121.71	120.30	H10A–C10–H10B	109.51	109.40
C4–C5–H5	119.13	119.19	H10A–C10–H10C	109.44	109.31
C6–C5–H5	119.16	120.51	H10B–C10–H10C	109.42	109.31

### Frontier molecular orbital (FMOs) and ultraviolet spectra analysis

**Fig. 6:**
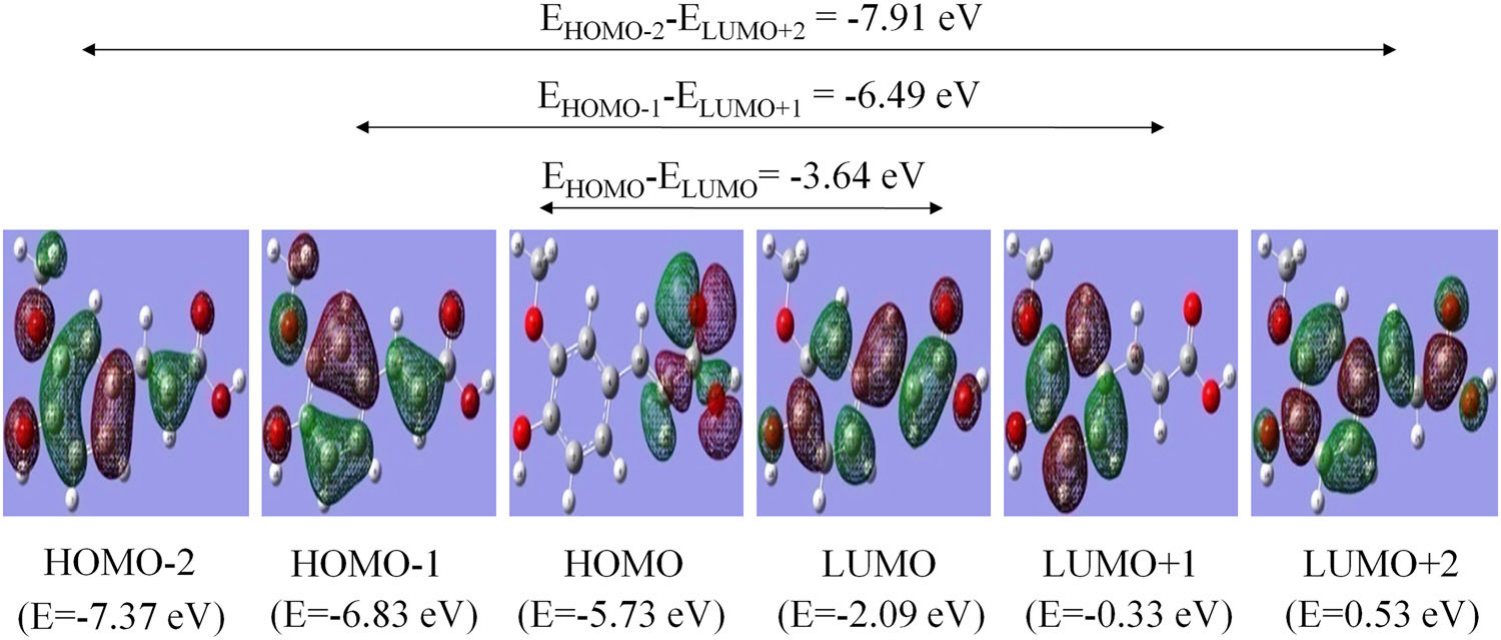
Molecular orbital surfaces and energy (eV) of FA for HOMO, HOMO-1, HOMO-2, LUMO, LUMO + 1 and LUMO + 2 in gas phase

Highest occupied molecular orbital (HOMO) and lowest unoccupied molecular orbital (LUMO) are known as frontier molecular orbital (FMOs), which plays a major role in the computation of UV–Vis spectra, electronic and optical properties of a molecule. HOMO represents the ability to donate an electron, while LUMO corresponds to the ability of a molecule to obtain an electron. The energy gap between these two orbitals helps to compute the physico-chemical properties viz. chemical hardness, chemical softness, kinetic stability, optical polarizability of the molecule. To evaluate the energetic behavior of FA, the calculations were done both in solution (ethanol and DMSO) and gas phase. The UV–Vis data for FA obtained by TD-DFT simulations have been complying with previously reported experimental results and found comparable (Asiri et al. 2011; Kosar and Albayrak 2011). The calculated values for the physico-chemical properties with the energies of the highest (HOMO), second highest (HOMO-1), third highest (HUMO-2), lowest (LUMO), second lowest (LUMO + 1) and third lowest (LUMO + 2) for FA are summarized in Table 5. These parameters are prerequisites for QSAR calculations of a compound. The molecular orbital diagrams for FA, computed at DFT/6-311G** basis set and shown in Fig. 6, confirmed that LUMO is localized on almost whole molecule, while HOMO is mainly localized on the side chain and near carboxylic acid. The values of energies for HOMO were calculated as −5.75, −5.76 and −5.73 eV, while for LUMO these values were computed as −2.28, −2.29 and 2.09 eV in ethanol, DMSO and gas phase, respectively. The energy gap between HOMO and LUMO also indicates the chemical stability of the molecule. The values of energy gap of FMOs in FA were found to be −3.46, −3.46 and −3.64 eV in ethanol, DMSO and gas phase, respectively. Theoretical absorption wavelength (*λ*), excitation energies (*E*) and oscillator strengths (*f*) for FA have been computed and given in Table 6. Data from theoretical calculations for physico-chemical properties of FA showed that the values are close in solution phase (ethanol and DMSO); while in gas phase, these are slightly deviated from those in solution phase.

**Table 5:** Calculated values of dipole moment, electronegativity, Chemical potential, chemical hardness, chemical softness, electrophilic index, and energies for ferulic acid in solution (ethanol and DMSO) and gaseous phase at TD-DFT/B3LYP/6-311G** basis set

TD-DFT/B3LYP/6-311G**	Ethanol	DMSO	Gas phase
Total energy (a.u)	−688.16	−688.16	−688.14
Dipole moment (Debye)	4.69	4.74	3.22
Chemical potential (eV)	−4.02	−4.02	−3.91
Chemical hardness (eV)	4.02	4.02	3.91
Chemical softness (eV)^−1^	0.12	0.12	0.13
Electronegativity (eV)	−1.74	−1.74	−1.82
Electrophilic index (eV)	0.376	0.375	0.423
E_HOMO_ (eV)	−5.75	−5.76	−5.73
E_HOMO-1_ (eV)	−6.89	−6.89	−6.83
E_HOMO-2_ (eV)	−7.66	−7.67	−7.37
E_LUMO_ (eV)	−2.28	−2.29	−2.09
E_LUMO+1_ (eV)	−0.28	−0.29	−0.33
E_LUMO+2_ (eV)	0.41	0.40	0.53
E_HOMO_–E_LUMO_	−3.46	−3.46	−3.64
E_HOMO-1_–E_LUMO+1_	−6.60	−6.60	−6.49
E_HOMO-2_–E_LUMO+2_	−8.07	−8.08	−7.91

**Table 6:** Absorption wavelength (*λ* in nm), excitation energies (*E* in eV) and oscillator strength (*f*) calculated at TD-DFT/B3LYP/6-311G** of ferulic acid

Ethanol			DMSO			Gas		
*λ* (nm)	*E* (eV)	*f*	*λ* (nm)	*E* (eV)	*f*	*λ* (nm)	*E* (eV)	*f*
403.43	3.0732	0.5568	404.94	3.0618	0.5681	363.53	3.4105	0.2598
311.01	3.9865	0.3866	311.67	3.9780	0.3877	300.86	4.1210	0.0000
293.76	4.2207	0.0000	293.60	4.2228	0.0000	292.11	4.2445	0.2743

### Analysis of docking results

**Fig. 7:**
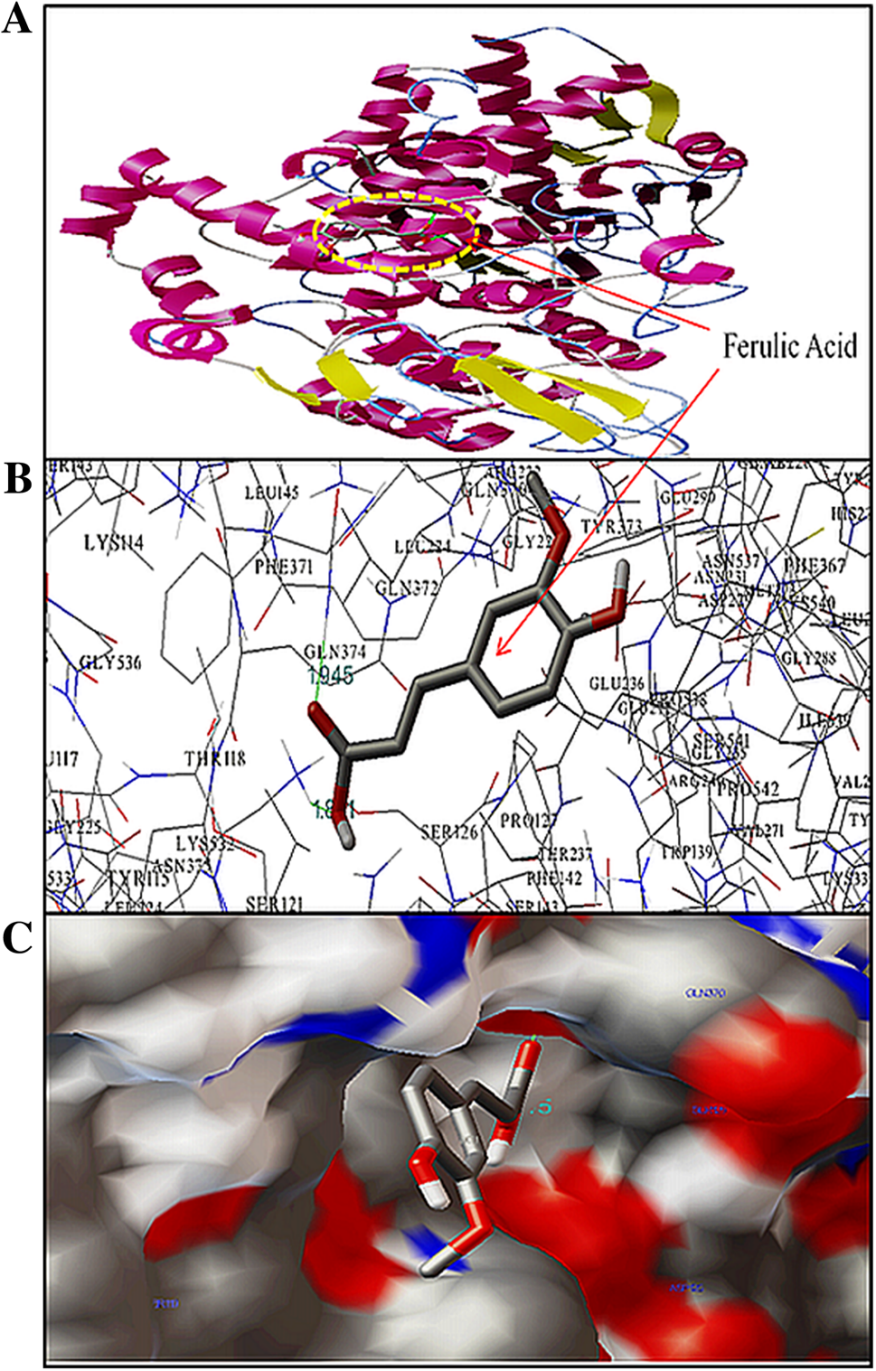
Docking of FA in the binding pocket of COX-2. Different view of FA and COX-2 docked conformation. **a** Overview in cartoon model of FA binding to COX-2, **b** the docking poses of the FA-COX-2 complex represented in a ball and stick model, and COX-2 is represented in line model, **c** hydrophobic pocket of COX-2 and FA, the FA represents in ball and stick model within the binding pockets in cyanocolor

All the docking poses of complex (COX-2 and FA) created by the AutoDock were loaded into PyMOL. The docking poses were ranked according to their docking scores and both the rank list of docked ligands with their corresponding binding poses have been given in Table S1. The analysis of the complex with the best docking conformation (with the least binding energy) showed that FA binds to the COX-2 mainly in the hydrophobic pocket. The stabilization of the FA-COX-2 complex has been carried out through the hydrogen bond interactions of different bond lengths between FA and amino acids of COX-2 enzyme. This study shows that the FA-COX-2 complex is stabilized by two hydrogen bonds of bond length 1.945 and 1.801 Å with the residues Gln372 and Lys532 of COX-2, respectively. The binding energy of the lowest energy conformer of FA-COX-2 complex was calculated computationally and found to be −5.25 kcal/mol. These results show the high affinity of FA with COX-2 enzyme. The binding pose of FA with COX-2 is illustrated in Fig. 7.

## Conclusions

This investigation started with the extraction and purification of FA from *P*. *hysterophorus* L. and its structural characterization has been carried out experimentally using different spectroscopic techniques and single crystal diffraction. FA was crystallized in the monoclinic space group (*P 21/n*) forming dimer via the formation of a strong hydrogen bond between –OH of –COOH group of one FA molecule with the oxygen atom of the C=O group of adjacent molecule and vice versa. A quantum chemical calculation for the optimization of FA geometry was done by Gaussian 09. The electronic and physico-chemical properties for the optimized geometry of FA have been also calculated, which showed degree of freedom, total energy and dipole moment to be 66, −688.012 Hartree and 2.50 Debye, respectively. The developed model comprises of geometry optimization at DFT/6-311G** basis set and the optimized geometry parameters and NMR chemical shifts of FA showed good correlation with the values obtained from X-ray crystal structure and experimental NMR. The calculated value for UV spectra of FA also matches with the experimental data. The theoretical as well as experimental data depicted structural resemblance of FA in solution and gaseous phase, which was confirmed by statistical significance of the data. The thermal studies carried out using non-isothermal TG-DTA-DTG inferred that FA undergoes single-step thermal decomposition. Further, molecular docking of FA with cyclooxygenase-2 was performed by AutoDock 4.2.3 program and we demonstrated that it binds with COX-2 enzyme in the hydrophobic pocket stabilized mainly by two hydrogen bonds. This study has tremendous scope in the pharmaceutical industry and biology for the designing of novel inhibitors for COX-2 enzyme.

## Electronic supplementary material

Below is the link to the electronic supplementary material.

Supplementary material 1 (DOCX 2532 kb)

Supplementary material the crystal structure of the FA was duly submitted in a databank with CCDC numbers 909271 which contains the supplementary crystallographic data (CIF) for this article. These data can be obtained free of charge from the Director, CCDC, 12 Union Road, Cambridge CB2 1EZ, UK (Fax: +44-1223-336-033; email: deposit@ccdc.cam.ac.uk). The TLC, HPLC, 3-D zig-zag view and table of energy of 30 docked conformations of protein–ligand complex are provided in the supplementary material.

## References

[CR1] Alam MA, Sernia C, Brown L (2013). Ferulic acid improves cardiovascular and kidney structure and function in hypertensive rats. J Card Pharm.

[CR2] Asiri AM, Karabacak M, Kurt M, Alamry KA (2011). Synthesis, molecular conformation, vibrational and electronic transition, isometric chemical shift, polarizability and hyperpolarizability analysis of 3-(4-Methoxy-phenyl)-2-(4-nitro-phenyl)-acrylonitrile: a combined experimental and theoretical analysis. Spectrochim Acta A.

[CR3] Becke AD (1993). Density functional thermochemistry. III. The role of exact exchange. J Chem Phys.

[CR4] Brandenburg K (1999) Diamond: visual crystal structure information system. Version 2.1c. Crystal Impact GbR, Bonn, Germany

[CR5] Chung SY, Champagne ET (2011). Ferulic acid enhances IgE binding to peanut allergens in Western blots. Food Chem.

[CR6] Ferguson LR, Shuotun Z, Harris PJ (2005). Antioxidant and antigenotoxic effects of plant cell wall hydroxycinnamic acids in cultured HT-29 cells. Mol Nutr Food Res.

[CR7] Frisch MJ et al. (2009) Gaussian 09, Revision A.1, Wallingford, CT

[CR8] Garg A, Manidhar DM, Gokara M, Malleda C, Reddy CS, Rajagopal Subramanyam R (2013). Elucidation of the binding mechanism of coumarin derivatives with human serum albumin. PLoS ONE.

[CR9] Goel N, Kumar N (2014). Study of supramolecular frameworks having aliphatic dicarboxylic acids, *N,**N*′-bis(salicyl)ethylenediamine and *N*, *N*′-bis(salicyl)butylenediamine. J Mol Struct.

[CR10] Goel N, Singh UP (2013). Syntheses, structural, computational, and thermal analysis of acid-base complexes of picric acid with *N*-heterocyclic bases. J Phys Chem A.

[CR11] Gunaseelan VN (1987). *Parthenium* as an additive with cattle manure in biogas production. Biol Waste.

[CR12] Kikugawa K, Hakamada T, Hasunuma M, Kurechi T (1983). Reaction of *p*-hydroxycinnamic acid derivatives with nitrite and its relevance to nitrosamine formation. J Agric Food Chem.

[CR13] Koopmans T (1934). About the allocation of wave functions and eigenvalues of the individual electrons one atom. Physica.

[CR14] Kosar B, Albayrak C (2011). Spectroscopic investigations and quantum chemical computational study of (E)-4-methoxy-2-[(*p*-tolylimino) methyl] phenol. Spectrochim Acta A.

[CR15] Kumar N, Bhalla TC (2011). In silico analysis of amino acid sequences in relation to specificity and physiochemical properties of some aliphatic amidases and kynurenine formamidases. J Bioinform Seq Anal.

[CR16] Kumar N, Pruthi V (2014). Potential applications of ferulic acid from natural sources. Biotechnol Rep.

[CR17] Maishi AI, Ali PKS, Chaghtai SA, Khan G (1998). A proving of *Parthenium hysterophorus* L. Brit Homoeopath J.

[CR18] Meng S, Cao J, Feng Q, Peng J, Hu Y (2013). Roles of chlorogenic acid on regulating glucose and lipids metabolism: a Review. Evid Based Complement Alternat Med.

[CR19] Middleton E, Kandaswami C, Theoharides TC (2000). The effects of plant flavonoids on mammalian cells: implications for inflammation, heart disease, and cancer. Pharmacol Rev.

[CR20] Mori H, Kawabata K, Yoshimi N, Tanaka T, Murakami T, Okada T, Murai H (1999). Chemopreventive effects of ferulic acid on oral and rice germ on large bowel carcinogenesis. Anticancer Res.

[CR21] Mori T, Koyama N, Guillot-Sestier MV, Tan J, Town T (2013). Ferulic acid is a nutraceutical β-secretase modulator that improves behavioral impairment and alzheimer-like pathology in transgenic mice. PLoS ONE.

[CR22] Morris GM, Huey R, Lindstrom W, Sanner MF, Belew RK, Goodsell DS, Olson AJ (2009). AutoDock4 and AutoDockTools4: automated docking with selective receptor flexibility. J Comp Chem.

[CR23] Ou S, Kwok KC (2004). Ferulic acid: pharmaceutical functions, preparation and applications in foods. J Sci Food Agric.

[CR24] Ou S, Li Y, Gao K (1999). A study on scavenging activity of wheat bran dietary fiber for free radical. Acta Nutr Sin.

[CR25] Patel S (2011). Harmful and beneficial aspects of *Parthenium hysterophorus*: an update. 3. Biotech.

[CR26] Peng C, Ayala PY, Schlegel HB, Frisch MJ (1996). Using redundant internal coordinates to optimize equilibrium geometries and transition states. J Comput Chem.

[CR27] Rajkumar EDM, Kumar NVN, Haran NVH, Ram NVS (1988). Antagonistic effect of *P. hysterophorus* on succinate dehydrogenase of sheep liver. J Environ Biol.

[CR28] Rosazza JPN, Huang Z, Dostal L, Volm T, Rousseau B (1995). Review: biocatalytic transformations of ferulic acid: an abundant aromatic natural product. J Industrial Microbiol.

[CR29] Sheldrick GM (1990). Phase annealing in SHELX-90: direct methods for larger structures. Acta Cryst.

[CR30] Sheldrick GM (1996). SADABS: program for empirical absorption correction of area detector data.

[CR31] Sheldrick GM (2000) SHELXTL-NT, Version 6.12, Reference manual, University of Gottingen, Germany

[CR32] Shorrock CJ, Rees WD (1988). Overview of gastroduodenal mucosal protection. Am J Med.

[CR33] Stephens PJ, Devlin FJ, Chablowski CF, Frisch MJ (1994). Ab initio calculation of vibrational absorption and circular dichroism spectra using density functional force fields. J Phys Chem.

[CR34] Tilay A, Bule M, Kishenkumar J, Annapure U (2008). Preparation of ferulic acid from agricultural wastes: its improved extraction and purification. J Agric Food Chem.

[CR35] Toshihiro A, Ken Y, Miho Y, Motohiko U, Yumiko K, Naoto S, Koichi A (2000). Triterpene alcohol and sterol ferulates from rice bran and their anti-inflammatory effects. J Agric Food Chem.

[CR36] Yang F, Zhou BR, Zhang P, Zhao YF, Chen J, Liang Y (2007). Binding of ferulic acid to cytochrome c enhances stability of the protein at physiological pH and inhibits cytochrome c-induced apoptosis. Chem Biol Interact.

[CR37] Zupfer JM, Churchill KE, Rasmusson DC, Fulcher RG (1998). Variation in ferulic acid concentration among diverse barley cultivars measured by HPLC and microspectrophotometry. J Agric Food Chem.

