# Supplementary material for: Structural elucidation and molecular docking of ferulic acid from Parthenium hysterophorus possessing COX-2 inhibition activity
Source: 3 Biotech. 2014 Oct 7;5(4):541–51. doi: 10.1007/s13205-014-0253-6 (PMC4522731; doi:10.1007/s13205-014-0253-6)
Supplement: Supplementary file 1 — Supplementary material 1 (DOCX 2532 kb) [file 13205_2014_253_MOESM1_ESM.docx]

**Supplementary material**

**Structural elucidation and molecular docking of ferulic acid from *Parthenium hysterophorus* possessing COX-2 inhibition activity**

Naresh Kumar and Vikas Pruthi*

Department of Biotechnology, Indian Institute of Technology Roorkee, Roorkee India-247667


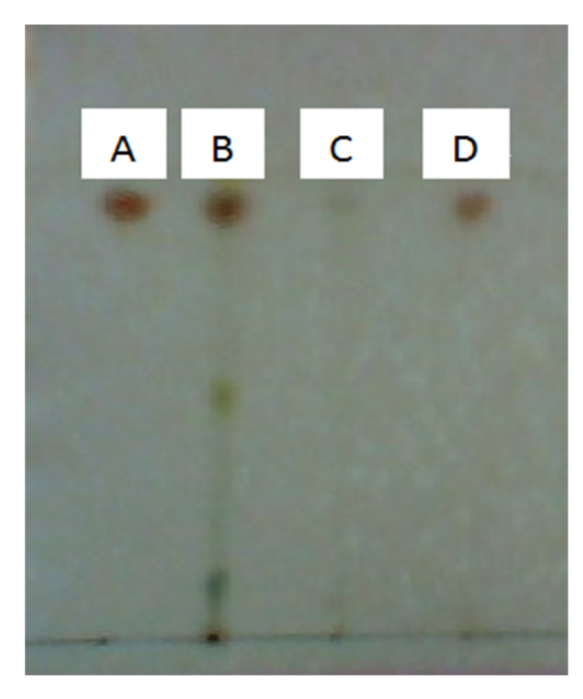


**Fig. S1:** Thin layer chromatoplate (TLC) of ferulic acid after spraying 10% ferric chloride reagent (A is control, B is stem extract, C is root extract and D is leaf extract of *P. hysterophorus* L.)


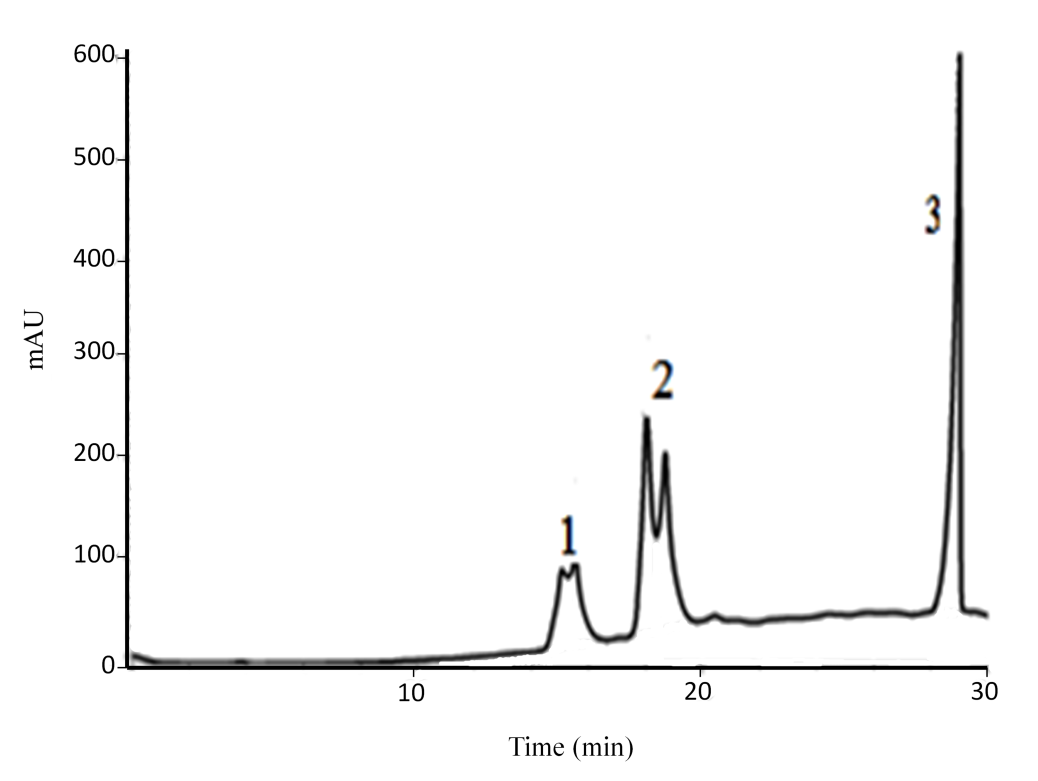


**Fig. S2:** HPLC profile of phenolic acids extracted from *P. hysterophorus* L. sample. Peaks 1, 2 and 3 were identified as caffeic acid, p-coumaric acid, and ferulic acid.

**
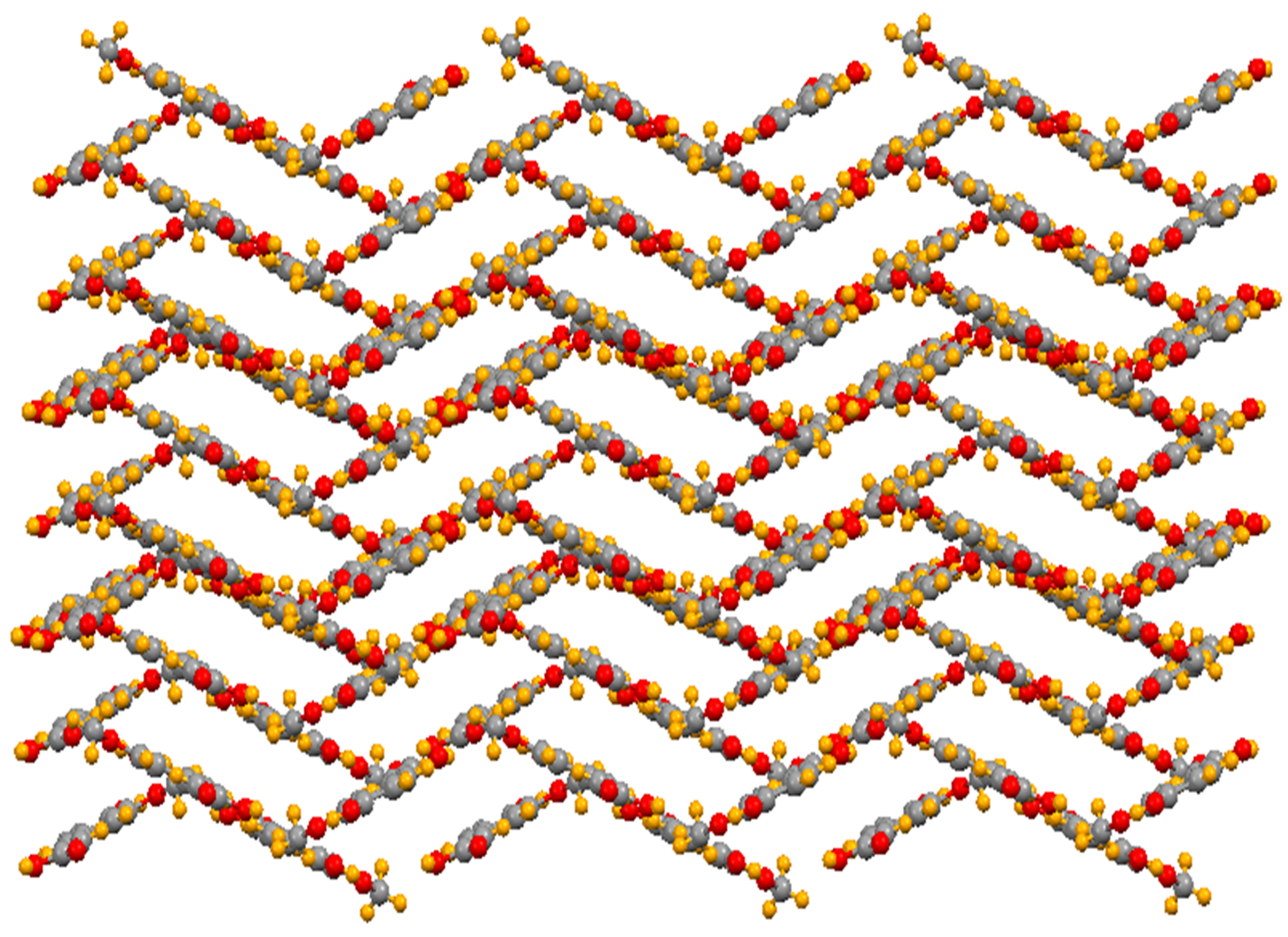
**

**Fig. S3:** Three dimensional zig-zag views in ferulic acid, representing the 3-D arrangement of atom present in ferulic acid.

**Table S1:** Docking Summary of cox-2 enzyme with ferulic acid generated different ligand conformers by the AutoDock4.2 program using the Lamarkian Genetic Algorithm.

| **Rank** | **Sub-Rank** | **Run** | **Binding Energy [Kcal M^-1^]** | **Inhibitory Constant, K_i_** |
| --- | --- | --- | --- | --- |
| 1 | 1 | 3 | -5.25 | 141.33µM |
| 1 | 2 | 21 | -5.18 | 158.52µM |
| 1 | 3 | 6 | -5.09 | 186.74µM |
| 1 | 4 | 16 | -5.05 | 199.78µM |
| 1 | 5 | 15 | -4.99 | 219.44µM |
| 1 | 6 | 9 | -4.97 | 226.97µM |
| 1 | 7 | 17 | -4.91 | 253.02µM |
| 1 | 8 | 7 | -4.83 | 287.70µM |
| 1 | 9 | 20 | -4.77 | 317.99µM |
| 1 | 10 | 22 | -4.67 | 379.28µM |
| 1 | 11 | 18 | -4.50 | 504.08µM |
| 1 | 12 | 29 | -4.29 | 712.22µM |
| 2 | 1 | 24 | -5.19 | 156.53µM |
| 3 | 1 | 25 | -5.18 | 159.09µM |
| 3 | 2 | 19 | -4.95 | 234.58µM |
| 3 | 3 | 10 | -4.67 | 374.64µM |
| 4 | 1 | 11 | -5.03 | 206.03µM |
| 5 | 1 | 26 | -5.00 | 215.12µM |
| 5 | 2 | 23 | -4.79 | 310.60µM |
| 6 | 1 | 2 | -4.85 | 277.95µM |
| 6 | 2 | 1 | -4.53 | 481.93µM |
| 7 | 1 | 5 | -4.74 | 333.68µM |
| 8 | 1 | 13 | -4.68 | 373.83µM |
| 8 | 2 | 27 | -4.51 | 490.99µM |
| 9 | 1 | 30 | -4.68 | 371.37µM |
| 10 | 1 | 28 | -4.67 | 376.82µM |
| 11 | 1 | 14 | -4.25 | 765.45µM |
| 12 | 1 | 12 | -4.12 | 960.38µM |
| 13 | 1 | 4 | -4.03 | 1.12mM |
| 14 | 1 | 8 | -3.50 | 2.70mM |

* The lowest free energy conformation is shown by the bold font which is the most stable conformation.
